# Thermal Behavior of Carbon-Phenolic/Silica Phenolic Dual-Layer Ablator Specimens through Arc-Jet Tests

**DOI:** 10.3390/ma16175929

**Published:** 2023-08-30

**Authors:** Rajesh Kumar Chinnaraj, Young Chan Kim, Seong Man Choi

**Affiliations:** Department of Aerospace Engineering, Jeonbuk National University, Jeonju-si 54896, Republic of Korea; rajkumchin@jbnu.ac.kr (R.K.C.); kyc0420@jbnu.ac.kr (Y.C.K.)

**Keywords:** thermal protection system, spacecraft heat shield, ablative materials, plasma wind tunnel, carbon phenolic, silica phenolic, atmospheric re-entry

## Abstract

We studied the behavioral characteristics of a newly developed dual-layer ablator, which uses carbon-phenolic as a recession layer and silica-phenolic as an insulating layer. The ablator specimens were tested in a 0.4 MW supersonic arc-jet plasma wind tunnel, employing two different shapes (flat-faced and hemispherical-faced) and varying thicknesses of the carbon-phenolic recession layer. The specimens underwent two test conditions, namely, stationary tests (7.5 MW/m^2^, ~40 s) and transient tests simulating an interplanetary spacecraft re-entry heat flux trajectory (6.25↔9.4 MW/m^2^, ~108 s). During the stationary tests, stagnation point temperatures of the specimens were measured. Additionally, internal temperatures of the specimens were measured at three locations for both stationary and transient tests: inside the carbon-phenolic recession layer, inside the silica-phenolic insulating layer, and at the recession layer–insulating layer intersection. The hemispherical-faced specimen surface temperatures were about 3000 K, which is about 350 K higher than those of flat-faced specimens, resulting in higher internal temperatures. The recession layer internal temperatures rose more exponentially when moved closer to the specimen stagnation point. Layer interaction and insulating layer internal temperatures were found to be dependent on both the recession layer thickness and the exposed surface shape. The change in exposed surface shape increased mass loss and recession, with hemispherical-faced specimens showing ~1.4-fold higher values than the flat-faced specimens.

## 1. Introduction

Thermal protection systems (TPS), also known as heat shields, are crucial components for any Earth return space missions. A TPS or a heat shield protects a spacecraft from extreme heat flux conditions exerted on it during its atmospheric re-entry. These extreme heat flux conditions are the result of a high thermal plasma flow generated due to the dissociation and ionization of air surrounding the spacecraft, caused by atmospheric drag and aerodynamic heating. The failure of TPS results in the failure of the entire space mission, thus making the TPS a single-point-of-failure (SPOF) subsystem [1].

Based on how they react during atmospheric re-entry, the TPS/TPS materials are classifiable into two categories: 1. reusable, non-ablative, and non-charring; 2. non-reusable and ablative, including charring (non-melting) and non-charring (melting) subtypes [2].

During the re-entry, reusable TPS materials are immune to physical and chemical changes, and their properties remain the same before and after the re-entry process. In general, reusable TPS can only withstand low heat flux conditions that occur during re-entries from low Earth orbit. Typically, during re-entry, a reusable TPS re-radiates a significant portion of the heat load and absorbs the rest; hence, reusable TPS is sometimes referred to as a ‘heat-soak TPS’. A classic example of a reusable TPS is reinforced carbon-carbon (RCC), which was used for NASA’s space shuttles [3].

The non-reusable charring TPS materials are go-to heat shield materials for interplanetary space missions and high-Earth-orbit re-entries. All NASA interplanetary space missions so far have used non-reusable ablative charring TPS materials [1]. Organic resins like phenolic along with solid re-enforcement phases are typical constituents of an ablative charring TPS material [4]. For phenolic-based ablators, when exposed to thermal loads, the pyrolysis or thermal degradation of the phenolic resin is an endothermic process that absorbs a portion of the heat; more details about the pyrolysis mechanism of phenolic resins can be found in Bessire et al. [5]. Additionally, the blockage effect caused by the pyrolysis gases on the surfaces exposed to the thermal loads reduces an amount of heat transferred to those surfaces. Due to these reasons, phenolic-based ablators are the most preferred choice for spacecraft heat shield applications when compared to other types of TPS materials. It is also interesting to note that the combined process of pyrolysis and char formation also plays an important role in some fire retardant materials [6]. A comprehensive review of various ablative materials for different thermal protection applications, including a compilation of widely used material testing techniques and ablation simulation codes, can be found in Natali et al. [7]. A one-dimensional thermal model for analyzing material behaviors during uncontrolled atmospheric re-entries of space debris, such as used rocket stages and phased-out satellites, can be found in Pirrone et al. [8].

Among the phenolic-based TPS materials, carbon-phenolic-based ablative materials (CPBAMs) are most commonly used for interplanetary Earth return space missions. Some examples of CPBAMs used for Earth return space missions include AVCOAT, used for the Apollo lunar missions [9]; PICA, used for the Stardust Wild 2 comet mission [10]; and MC-CFRP, used for the Hayabusa Itokawa asteroid mission [11]. CPBAMs are the current industrial norm for high-profile space missions; for example, AVCOAT is once again being used on Orion spacecraft as a part of the Artemis moon mission program [12] and PICA-X, a material similar to PICA is used for SpaceX’s Dragon class spacecraft [13]. Besides spacecraft TPS applications, CPBAMs are also used in solid propellant rocket motor nozzles, such as those used in NASA’s space shuttle programs [14] and surface-to-air missiles [15]. Despite being excellent ablators, carbon-phenolic materials are considered poor thermal insulators [1]. The poor thermal insulation, which is a result of high thermal conductivity, makes carbon-phenolic materials favorable for enhancing heat dissipation in electronic components [16]. However, for spacecraft heat shield applications, the poor thermal insulation of carbon-phenolic materials is unfavorable. Therefore, it is necessary to enhance their thermal insulation properties in order to protect the metallic frame of re-entry spacecraft, as common high-temperature resistant aluminum alloys begin to lose their strength when the temperature exceeds ~473 K (i.e., 200 °C) [17]. So, in principle, it is important to design the spacecraft heat shield to maintain its back face temperature below ~473 K (i.e., 200 °C) during atmospheric re-entry. This is necessary to prevent any substantial loss of strength in the spacecraft’s metallic structure, considering the extremely high forces experienced during the re-entry phase. The lower thermal insulation provided by the TPS material will result in a thicker spacecraft heat shield, which increases the overall mass of the spacecraft. For future Korean spacecraft applications, a nominal value of 453.15 K (i.e., 180 °C) is designated as the maximum heat shield back face temperature, i.e., as a design limit.

It is possible to modify the overall thermal properties of a carbon-phenolic material to meet specific application requirements, as the thermal properties of carbon-phenolic materials depend on the composition of their constituent materials and their structural arrangement. In CPBAM heat shields, one method to enhance the overall thermal insulation is by reducing the density of carbon-phenolic materials through alterations in their internal microstructures and composition. This reduction effectively decreases thermal conductivity, thereby increasing thermal insulation. However, it should be noted that this process also leads to a decrease in material strength [18]. This method warrants a careful understanding of carbon-phenolic material properties; however, most of the current methods for measuring carbon-phenolic material properties are considered outdated, necessitating the development of new measurement techniques [19].

Another method to improve the overall thermal insulation provided by a CPBAM heat shield is by adding an inner secondary insulating layer with a lower thermal conductivity to an outer primary flow-facing recession layer [20].

In this experimental study, the tested ablative material (henceforth referred to as ‘ablator’) is developed based on the second method, where an outer primary carbon-phenolic recession layer is augmented with an inner secondary silica-phenolic insulating layer, thus constituting the dual-layer ablator. This dual-layer ablator is developed based on our two previous studies [21,22]. In this ablator, the average ratio of temperature-wise thermal conductivity, measured up to 773.15 K (i.e., 500 °C) of the ablator’s silica-phenolic material, is 1/3.5 of that of the carbon-phenolic material [22].

Since the ablator is developed using carbon-phenolic material, and the ablation process is confined to it (as confirmed in the previous study [22]), owing to its role as the primary flow-facing recession layer, the dual-layer ablator is classified as belonging to the CPBAM category of TPS materials.

### 1.1. Background

In the first of our two previous studies [21], we conducted a preliminary investigation of two sets of TPS material specimens. The first set consisted of carbon-phenolic specimens with two different lamination angles of 0° and 30°. And the second set consisted of two specially designed SiC-coated carbon–carbon composite specimens, one with a cork base and the other with a graphite base. This preliminary investigation was carried out using JBNU’s HVOF (high-velocity oxygen fuel) material ablation test facility [23,24]. The specimens were exposed to heat flux test conditions ranging from 3.25 to 11.5 MW/m^2^. The heat flux test conditions were selected to correspond to an interplanetary spacecraft’s re-entry heat flux trajectory. Based on the overall test results, we selected carbon-phenolic material with the 30° lamination angle (henceforth referred to as ‘30° carbon-phenolic’) for further development. Nevertheless, the 30° carbon-phenolic material’s internal temperatures measured during the tests did not fully satisfy the design limit criteria of 453.15 K (i.e., 180 °C), warranting further improvement. In the same study, it was also noted that the cork internal temperature values of the SiC-coated carbon–carbon composite specimen with the cork material base trended slightly above or closer to the design limit of 453.15 K (i.e., 180 °C). For the same specimen, during the test, the cork material section was charred and detached from the SiC-coated section at the carbon adhesive layer, which was used to bond the two sections of the specimen.

In the follow-up study [22], two different dual-layer ablators were developed. Based on the observations made in the preceding study [21], one of the ablators was developed by adding the cork material as the inner insulating layer to the 30° carbon-phenolic material. Cork and cork-based materials find their application in various TPS applications [10,25,26], as cork is known for its low density, high compressibility, resilience to vibration, excellent stability, and low thermal conductivity; its low thermal conductivity is due to the air confined within its structure [27].

In the same study [22], another ablator was developed by adding a silica-phenolic material as the inner insulating layer to the 30° carbon-phenolic material. The ablation processes of silica-phenolic materials have also been extensively studied due to their TPS applications [28,29,30,31,32]. Two prominent examples of silica-phenolic ablators used as primary spacecraft heat shield materials are Aleastrasil [33], used in the European Space Agency’s (ESA) Atmospheric Reentry Demonstrator (ARD), and AQ60/I [34] for the ESA’s Huygens mission to Saturn’s moon of Titan.

Ablation tests of the ablator specimens in Chinnaraj et al. [22] were conducted using JBNU’s 0.4 MW supersonic arc-jet plasma wind tunnel (PWT) [35,36]. A comprehensive comparison of various atmospheric re-entry stimulating PWTs can be found in Leohle et al. [37]. A recent review on computational and experimental modeling of plasma flows can be found in Kuzenov et al. [38]. In Chinnaraj et al. [22], two test conditions were employed; a stationary test condition and a transient test condition. First, in stationary tests, the specimens were exposed to the plasma test flow at a heat flux of 7.5 MW/m^2^ for a duration of 50 s. Stationary test conditions, i.e., where specimens were exposed to the test flow at a constant heat flux for a certain duration of time, are the norm in similar studies [4,13,18,20,39,40,41,42,43,44,45]. In Chinnaraj et al. [22], stationary tests were conducted for the preliminary investigation of the structural integrity of dual-layer ablator specimens, as the outer recession layer and inner insulating layer were mechanically bonded together. The visual inspection conducted after the stationary tests revealed that the specimens remained structurally intact; no separation or gaps were found between the layers. But for the carbon-phenolic/cork specimen, smoke was seen emanating from the specimen after the stationary test. The possible reason for the smoke emitted from the carbon-phenolic/cork specimen is considered to be the evaporation of water content in the cork, which could result in the shrinkage of the cork layer and potentially cause significant damage to the structural integrity of the spacecraft during re-entry. As the exact reasons for smoke emission and its repercussions were not precisely determined, the carbon-phenolic/cork specimen was not subjected to further tests (i.e., the transient tests) in Chinnaraj et al. [22] and is also not being considered for further development for the Korean spacecraft applications. In Chinnaraj et al. [22], the transient test condition was selected to simulate a spacecraft’s re-entry heat flux trajectory, where the carbon-phenolic/silica-phenolic ablator specimens, while being exposed to test flow, transitioned from a heat flux position of 6.25 MW/m^2^ to a heat flux position of 9.4 MW/m^2^ and back for an approximate duration of 110 s. The transient test condition was used because in real ballistic re-entry, the heat flux experienced by a spacecraft varies over time, i.e., transient in nature. During a ballistic re-entry, the heat flux experienced by the spacecraft is zero prior to the point of re-entry. It gradually increases to a maximum value within the atmosphere and then decreases until it reaches zero again before the spacecraft lands on the ground. Therefore, it is essential to subject candidate materials for spacecraft heat shield applications to transient time-varying heat flux test conditions in order to obtain more accurate material responses. It is also important to note that studies utilizing transient heat flux conditions are very rare. One study that could be found is Auweter-Kurtz et al. [46], where the peak heat flux was 2.02 MW/m^2^, which is much lower than the expected peak heat flux value in the re-entry trajectory of an interplanetary spacecraft. In Chinnaraj et al. [22], the transient tests showed that the carbon phenolic/silica phenolic ablator specimens are able to maintain their internal temperatures at the measured locations below the targeted design limit of 453.15 K (180 °C) when exposed to heat fluxes expected during an interplanetary spacecraft’s re-entry heat flux trajectory. As a result of these findings, the carbon-phenolic/silica-phenolic ablator (henceforth referred to as ‘CP/SP ablator’) has been chosen as the preferred TPS material for future Korean spacecraft applications.

Even though previous study [22] results are promising, it is important to subject the CP/SP ablator to various configurations and conditions in order to carefully evaluate its performance before its application in an actual spacecraft heat shield.

### 1.2. Objective

The objective of the current study is to analyze the ablation reaction of the CP/SP ablator specimens under different structural configurations. The methodology for this study is similar to the one used in the previous study [22] including the tested heat fluxes, but the number of specimens tested is three times greater. A total of 12 specimens were tested for this study, while only four specimens were tested in the previous study [22]. Here, in the current study, the tested ablators had different thicknesses of the carbon-phenolic recession layer, with either a flat or hemispherical exposed surface (i.e., the surface that is exposed to the plasma test flow). This was carried out to understand the following aspects:

1. How does the variation in outer recession layer thickness influence the ablator internal temperatures?

2. In hypersonic vehicles like re-entering spacecraft, the shape of the exposed surface influences the stagnation point heating [47], thereby affecting the stagnation point temperature. Considering this, to what extent is the ablator specimen’s stagnation point temperature influenced by the shape of the specimen’s exposed surface in the PWT tests? Furthermore, to what extent does the shape of the specimen’s exposed surface influence the ablator internal temperatures?

The first aspect will help optimize the ratio between the recession layer thickness and the insulating layer thickness of the CP/SP ablator, while the second aspect will help to gain insights on how to design or optimize the overall spacecraft heat shield shape by knowing how different ablator shapes react differently to identical heat flux test conditions. The results obtained from this study will greatly influence the final design of a future Korean spacecraft.

## 2. Materials and Methods

### 2.1. Specimens

The manufacturing process of the carbon-phenolic material (final density ≈ 1340 kg/m^3^) is as follows: first, a bulk carbon-phenolic block was fabricated by impregnating a rayon-based carbon fabric with a resol phenolic resin, followed by vacuuming and hydro-clave processes. The desired lamination angle of 30° was achieved by cutting the processed bulk carbon-phenolic block using a hole cutter at a 30° angle.

The manufacturing process of the silica-phenolic material (final density ≈ 1791 kg/m^3^) used as the inner insulating layer for the ablator specimens was similar to that used for the carbon-phenolic material. The manufacturing process of the silica-phenolic material began with the impregnation of an arranged stack of silica sheets with the resol phenolic resin, followed by vacuuming and hydro-clave processes. Then, the processed bulk silica-phenolic block was cut using the same hole cutter used for the carbon-phenolic material to obtain a lamination angle of 30°. Thus, the lamination angle of both carbon-phenolic and silica-phenolic materials in the CP/SP ablator is 30°.

As mentioned earlier, a total of 12 CP/SP ablator specimens were tested, with 6 being flat-faced specimens and the remaining 6 being hemispherical-faced specimens. The 6 flat-faced specimens consisted of three pairs, each pair having the same thickness of the carbon-phenolic recession layer. Within each pair, one specimen was subjected to the stationary test, while the other specimen was subjected to the transient test. This same arrangement was also applied to the hemispherical-faced specimens. The thickness of each specimen’s silica-phenolic insulating layer was 20 mm, with a diameter of 30 mm. The recession layer of each specimen had a lower stem section with a thickness of 15 mm and a diameter of 30 mm, to which the insulating layer was connected at the bottom. In the flat-faced specimens, the top section of the recession layer in each specimen had a diameter of 50 mm, which was exposed to the plasma test flow. The thickness of the top section varied with values of 10 mm, 15 mm, and 20 mm, with two specimens having the same thickness, resulting in a total of 6 flat-faced specimens.

In the case of the hemispherical specimens, most of the dimensions of the hemispherical specimens were the same as those of the flat-faced specimens, except that each hemispherical-faced specimen had an additional hemispherical or ‘blunt nose-cone’-like section on the top, with a ‘nose radius’ of 25 mm and a thickness of 11.88 mm. This hemispherical section was designed to mimic the nose-cone part of a spacecraft heat shield. In the hemispherical specimens, the total thickness of the recession layer varied by adjusting the thickness of the middle section of the recession layer, with values of 10 mm, 15 mm, and 20 mm. Two specimens were fabricated for each thickness, resulting in a total of 6 hemispherical-faced specimens.

In the specimens, the recession layers and insulating layers were attached to each other using commercially available steel M3 bolts that were 25 mm long, along with 3 mm thick aluminum washers. Two bolts were used for each specimen, with one aluminum washer per bolt. The bolt drill holes were sealed with silica-phenolic material after bolting the carbon-phenolic recession layers and silica-phenolic insulating layers together.

### 2.2. Experimental Setup

JBNU’s 0.4 MW supersonic segmented-type arc-jet PWT was used for this study. The PWT contains a gas supply manifold, segmented-type arc plasma torch, vacuum test chamber, diffuser, heat exchanger, cooling water supply, DC power supply, and vacuum pump system. The PWT can generate plasma test flows either in the range of Mach 2 or Mach 3, depending on the plasma torch convergent-divergent nozzle dimensions. The Mach 2 plasma torch nozzle was used for this study, and the nozzle’s exit diameter is 16 mm, while the throat diameter is 10.6 mm. A water-cooled four-armed displacement mechanism located inside the vacuum test chamber is useful for mounting specimens and other intrusive flow diagnostics probes or tools and exposing them to plasma test flow. The displacement mechanism is remotely controlled and can be rotated and moved in parallel and perpendicular to the plasma test flow. The vacuum test chamber has visual ports, which allow for visual observation of the tests and facilitate various optical measurements like pyrometry and spectrometry.

Prior to each test, the specimen was placed on the displacement mechanism, the vacuum test chamber was sealed, and cooling water circulated to the PWT components. Then, a low-pressure condition inside the vacuum test chamber was obtained by operating the vacuum pumps. Once the desired low chamber pressure was reached, a stream of argon gas was introduced into the plasma torch. The combination of high injection pressure and low chamber pressure directed the argon stream towards the test chamber. Afterward, high DC power was applied to the plasma torch’s dual pairs of electrodes. As a result, an electric arc was struck between the electrode pairs through the argon stream. The working gas mixture of air and argon was supplied to the plasma torch via its constricted segmented packs. Inside the constricted space of the plasma torch, the working gas and the generated electric arc interacted with each other and underwent high thermal exchange, causing N_2_ and O_2_ molecules in the working gas mixture to dissociate and ionize, forming hot air plasma. By virtue of the high pressure difference between the plasma torch and the test chamber, the hot air plasma exited the plasma torch’s convergent-divergent nozzle as a supersonic high enthalpy plasma flow, to which the specimen was exposed. More details on JBNU’s 0.4 MW supersonic arc-jet PWT operating procedures, specifications and schematics can be found in [35,36].

Table 1 shows the PWT operating conditions used for this study [22].

Earlier, prior to specimen tests, a water-cooled Gardon gauge was used to measure the PWT test flow’s stagnation point cold wall heat flux in the flow’s axial direction under the operating conditions mentioned in Table 1.

The main purpose of the stationary tests in this study was to understand how the shape of the specimen surface exposed to the test flow influences the specimen stagnation point temperature. A two-color pyrometer (IMPAC series ISQ 5 MB 14 model from LumaSense Technologies, Sanat Clara, CA, USA, with a measurement range from 1273.15 K to 3273.15 K) was used to measure the specimen stagnation point temperatures during the stationary tests. The pyrometer was installed at a fixed location on one of the visual ports of the PWT test chamber and was not flexible enough to be moved; only limited adjustments were possible prior to each stationary test. Therefore, the pyrometer could not be used for transient tests when the specimens were in motion. This is why stationary tests were necessary to measure the stagnation temperatures of the specimens. Each stationary test was conducted at 7.5 MW/m^2^ for ~40 s, where the specimen was exposed to the plasma test flow at a distance of 170 mm from the plasma torch nozzle exit. The stationary test heat flux value used in this study is the same as that in the previous study [22], except in the current study, the stationary test duration was shortened to ~40 s instead of the previously used 50 s, as the purpose of the stationary tests is different in the current study compared to the previous study.

For transient tests, each specimen was inserted into the plasma test flow at a distance of 180 mm from the plasma torch nozzle exit while still being exposed to the test flow, and the specimen was moved to a location 120 mm from the nozzle exit for a duration of 50 s. Once the specimen reached the distance of 120 mm from the nozzle exit, there was a delay of approximately 8 s (averaged from all transient tests) to reset the PWT displacement mechanism to reverse its direction of motion and move the specimen back to the original location of 180 mm from the nozzle exit. This reverse motion took another 50 s. Therefore, in the transient tests, each specimen’s total exposure time to the test flow was approximately 108 s. The duration of the transient test in this study was approximately 2 s shorter than that of the previous study [22], as efforts were made to reduce the motion reset time of the PWT displacement mechanism. Also, like the stationary tests, the transient test heat flux values are the same as those in the previous study [22]. The heat flux value at the 120 mm distance was measured prior using the Gardon gauge as 9.4 MW/m^2^. For the 180 mm distance, the heat flux value of 6.25 MW/m^2^ was determined empirically, using previously obtained Gardon gauge measurements. The empirical estimation of heat flux value at 180 mm was needed due to the inoperability of the PWT’s Gardon gauge due to some damages sustained during the earlier measurement experiments.

Thus, the maximum and minimum heat flux values used for transient tests are 9.4 MW/m^2^ and 6.25 MW/m^2^, respectively, which corresponds to the high-peak portion of an interplanetary spacecraft’s re-entry heat flux trajectory (6.25 MW/m^2^ → 9.4 MW/m^2^ → 6.25 MW/m^2^). It is also important to note that the heat flux value of 7.5 MW/m^2^ was selected for the stationary tests, as this value is closer to the average of the maximum and minimum heat flux values of the transient tests.

Table 2 summarizes the specimen test conditions.

The internal temperatures of the specimens were measured for both the stationary and transient tests. For each specimen, three K-type thermocouples were used to measure the internal temperatures at distances of 30 mm, 20 mm, and 10 mm (henceforth referred to as TC1, TC2, and TC3, respectively) from the bottom surface of the specimen. In each specimen, three thermocouple slots were drilled in a straight line between the specimen’s two bolts, with the center slot being TC3. In each specimen test, TC2 was used to measure the internal temperature at the intersection of the carbon-phenolic recession layer and silica-phenolic insulating layer. TC1 was used to measure the internal temperature of the recession layer, thus positioned 10 mm above the recession layer–insulating layer intersection. TC3 was used to measure the internal temperature of the insulating layer, thus positioned 10 mm below the recession layer-insulating layer intersection. This arrangement was made to understand how the thermal responses of two layers of the CP/SP ablator vary with different recession layer thicknesses and the shape of the surface exposed to the plasma test flow. The thermocouple slots were each 1.6 mm in diameter and fitted with thermocouples of a diameter of 1.57 mm. The distance between the center of each thermocouple slot was 3 mm. The thermocouple data acquisition system used in this study consisted of a National Instruments NI cDAQ-9178, which is a compact data acquisition USB chassis, along with an NI 9212 thermocouple input module.

**Figure 1:**
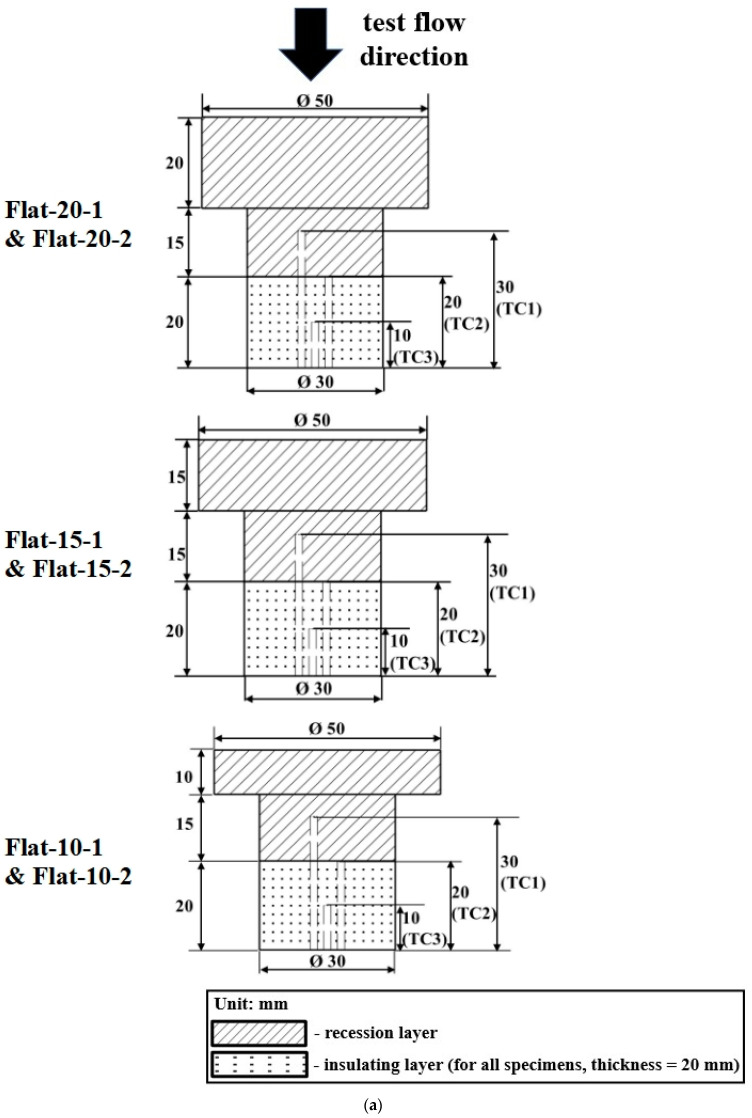

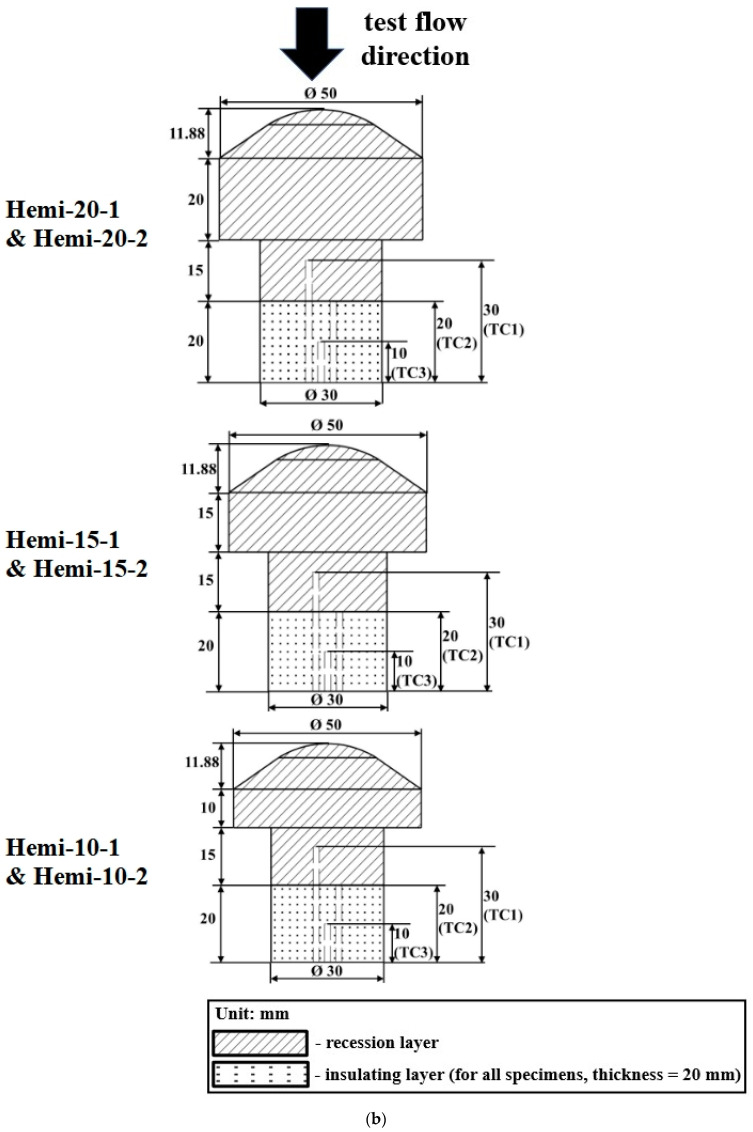
(**a**) Specimen dimensions (flat-faced). (**b**) Specimen dimensions (hemispherical-faced).

Figure 1a,b show the specimen dimensions, where internal connecting bolts are not shown for clarity.

Table 3 lists each specimen along with the test condition, total length, recession layer thickness, and locations of thermocouple measurements from the stagnation point.

**Figure 2:**
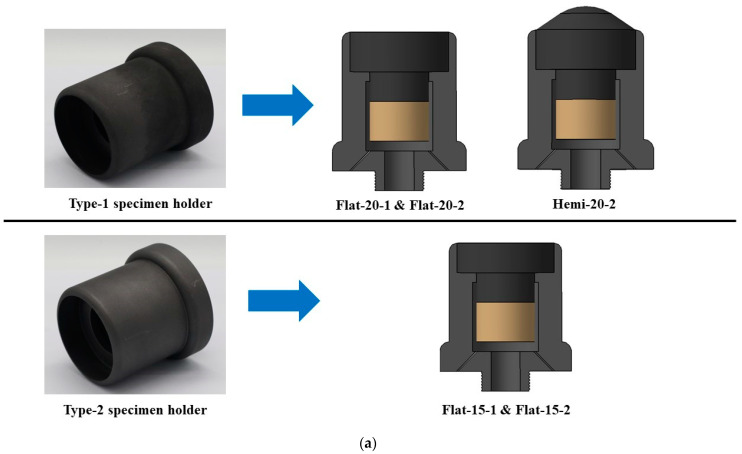

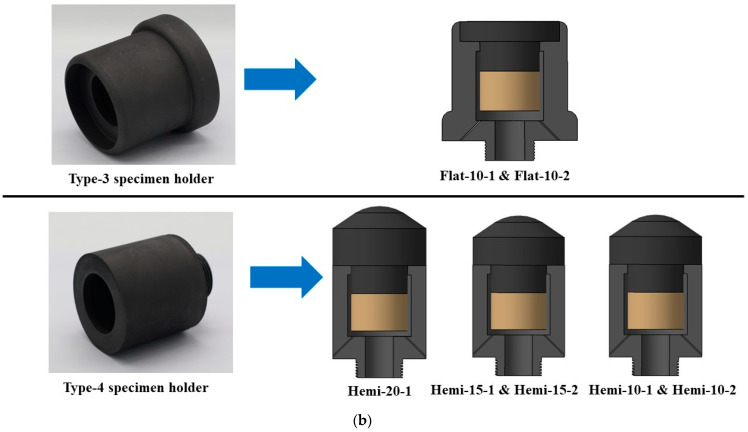
(**a**) Specimen holders (type-1 and type-2). (**b**) Specimen holders (type-3 and type-4).

During the tests, the specimens were installed in specimen holders before mounting to the PWT displacement mechanism. The specimen holders were made of graphite in order to reduce heat transfer in the specimen's lateral directions. The reduction in heat transfer in lateral directions is one of the reasons for each specimen’s diameter being approximately 3-fold the diameter of the plasma test flow. Another reason was to avoid ablation on the specimen’s sides, confining mass loss and recession only to the exposed surface. Figure 2a,b show the specimen holders along with illustrations of corresponding specimens used with them during the tests. Initially, only three types of specimen holders (type 1, 2, and 3) were fabricated. However, during the transient test of the Hemi-20-2 specimen, a design flaw was identified in type 1, 2, and 3 specimen holders (further discussed in Section 3). Subsequently, a modified specimen holder (type-4) was used for the remaining tests of hemispherical-faced specimens. Each specimen holder was fabricated with two ventilation holes to allow air trapped within the specimen holder to expand caused by high temperatures during the test without damaging the specimen holder.

A 3-D optical/non-contact VR-5200 measurement system was used to obtain three-dimensional images of each specimen’s exposed surface after the test in order to study the surface morphological changes caused by the ablation.

**Figure 3:**
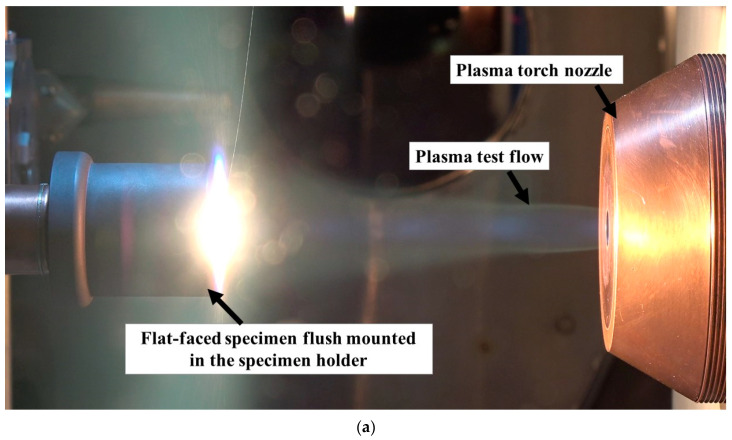

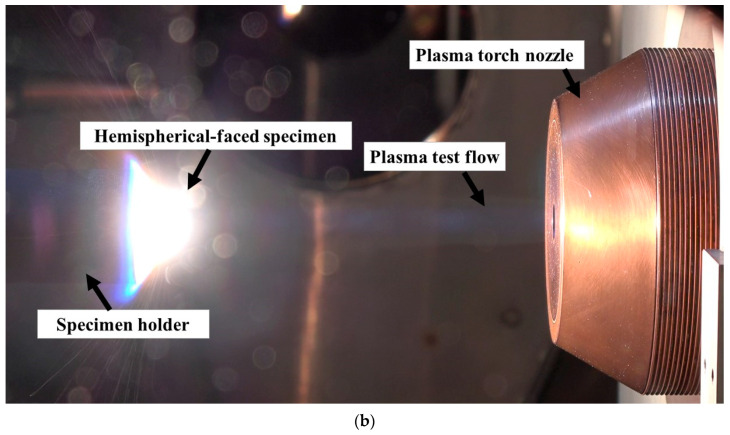
(**a**) Flat-faced specimen test photograph (Flat-20-1). (**b**) Hemispherical-specimen test photograph (Hemi-20-1).

Figure 3a,b show photographs of specimens exposed to the plasma test flow during the tests.

**Figure 4:**
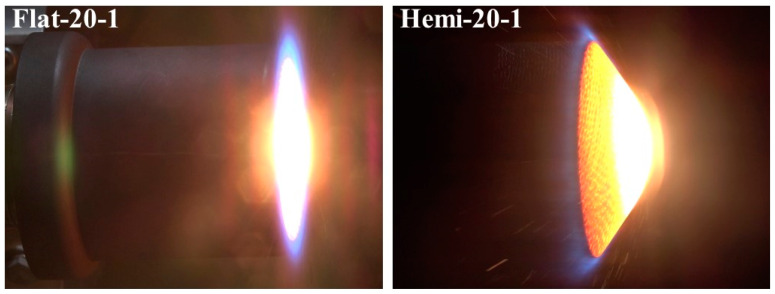
Specimen tests close-up photographs.

Figure 4 shows close-up photographs of a flat-faced specimen and a hemispherical-faced specimen being exposed to the plasma test flow, and the photographs clearly show how these two shapes interact differently with the supersonic plasma test flow.

## 3. Results and Discussion

In this section, the results are discussed in the order of the conducted experiments.

### 3.1. Flat-Faced Specimen Stationary Tests

**Figure 5:**
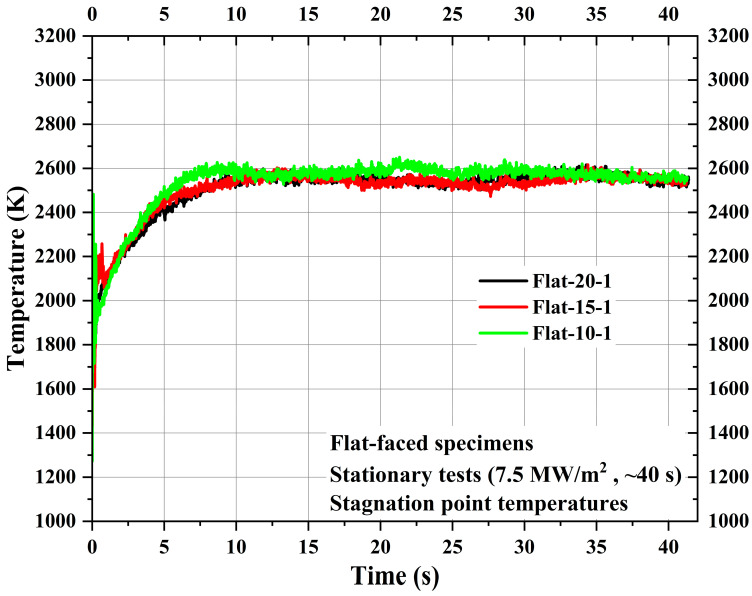
Flat-faced specimen stationary test stagnation point temperatures.

First, three flat-faced specimens, namely, Flat-20-1, Flat-15-1, and Flat-10-1, were subjected to stationary tests, where each specimen was exposed to 7.5 MW/m^2^ for ~40 s. Figure 5 shows the comparison of the stagnation point temperatures of the specimens Flat-20-1, Flat-15-1, and Flat-10-1. The stagnation point temperature responses from all three specimens were similar, as expected due to the identical test conditions, material, and shape of the exposed surface. The maximum stagnation point temperature values measured for the specimens Flat-20-1, Flat-15-1, and Flat-10-1 are 2616.25 K, 2616.65 K, and 2651.05 K, respectively. Similar values were also obtained in the previous study [22], where flat-faced specimens with the same carbon-phenolic recession layers were subjected to 7.5 MW/m^2^. This conclusively indicates that at 7.5 MW/m^2^, for the flat-faced shape, the 30° carbon-phenolic material attains a maximum surface temperature of around 2600 K.

**Figure 6:**
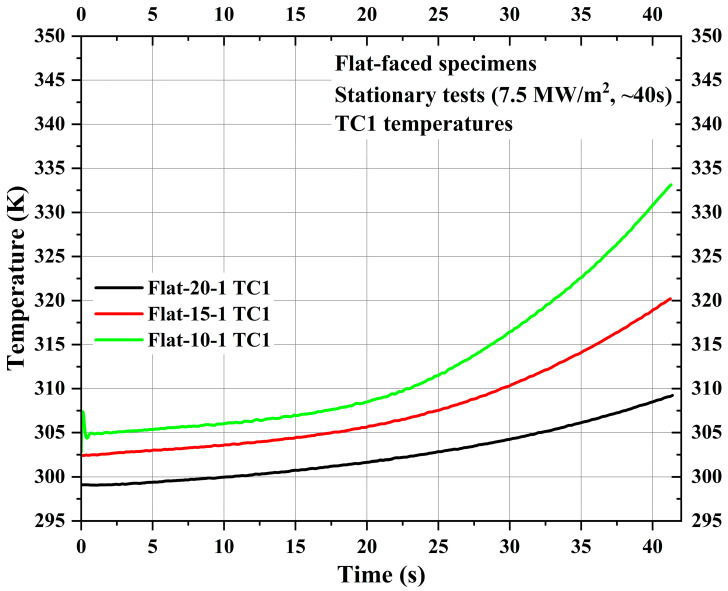
Flat-faced specimen stationary test TC1 temperatures.

Figure 6 shows TC1 measurements for the specimens Flat-20-1, Flat-15-1, and Flat-10-1. Though the location of TC1 was the same for all specimens from each specimen’s bottom surface, i.e., 30 mm, the respective locations of TC1 from each specimen’s stagnation point were 25 mm, 20 mm, and 15 mm for specimens Flat-20-1, Flat-15-1, and Flat-10-1, respectively, due to the variation in recession layer thickness. As TC1 was located inside the carbon-phenolic recession layer, Figure 6 shows that the internal temperature of the recession layer increases more exponentially when the measurement location moves closer to the stagnation point. This increased exponential trend may be attributed to ablative mass loss and recession occurring during the test, as specimen Flat-10-1’s TC1 was located closer to the receding exposed surface than in the other two specimens. The comparison shows that for the 10 mm difference between measurement locations, the maximum TC1 temperature difference was ~24 K, and for the 5 mm difference, the maximum average TC1 temperature difference was ~12 K.

**Figure 7:**
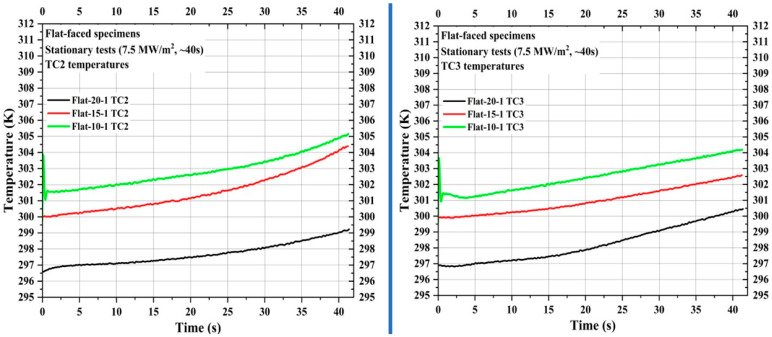
Flat-faced specimen stationary test TC2 and TC3 temperatures.

Figure 7 shows TC2 and TC3 measurements of the specimens Flat-20-1, Flat-15-1, and Flat-10-1. TC2 locations of the specimens Flat-20-1, Flat-15-1, and Flat-10-1 were 35 mm, 30 mm, and 25 mm, respectively, from the specimen stagnation point, while TC3 locations were 45 mm, 40 mm, and 35 mm, respectively, from the specimen stagnation point. Figure 7 shows that the TC2 and TC3 temperature values are almost identical to each other within the same specimen. Figure 7 shows that there was only a negligible difference of approximately 2 to 5 K between the initial and end of the test flow exposure TC2 and TC3 temperatures for the specimens Flat-20-1, Flat-15-1, and Flat-10-1. This indicates the good and remarkable performance characteristics of the CP/SP ablator.

**Figure 8:**
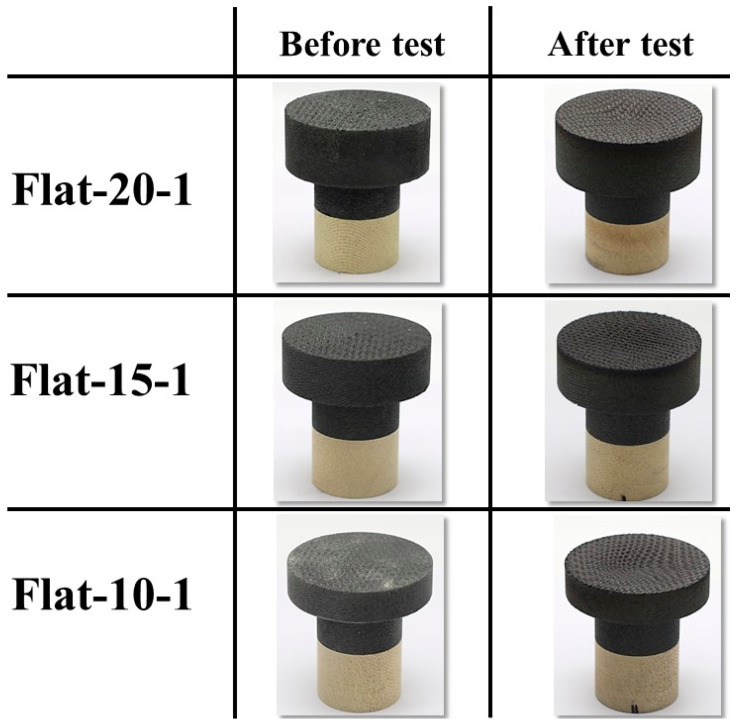
Flat-faced specimen stationary tests (7.5 MW/m^2^, ~40 s) before- and after-test images.

**Figure 9:**
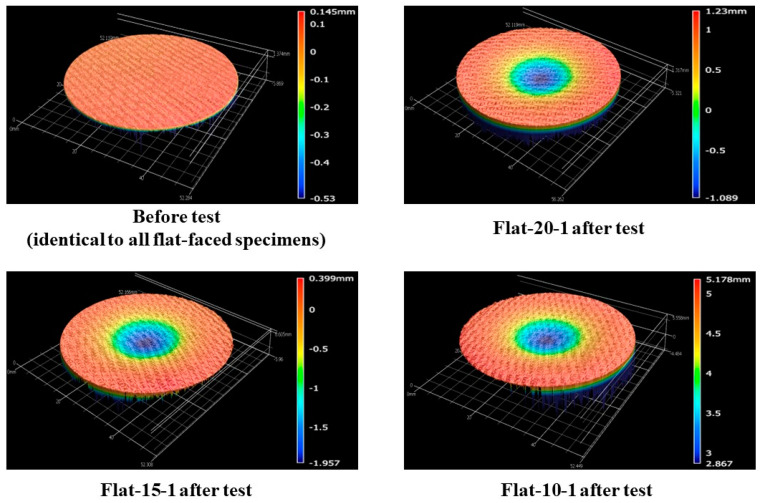
Flat-faced specimen stationary test (7.5 MW/m^2^, ~40 s)-exposed surface morphology changes.

Figure 8 shows the before- and after-test images of the specimens Flat-20-1, Flat-15-1, and Flat-10-1. Figure 9 shows the after-test-exposed surface morphologies of the specimens Flat-20-1, Flat-15-1, and Flat-10-1, compared with a before-test flat-faced specimen surface morphology. These images were taken using the three-dimensional optical/non-contact measurement system (i.e., the VR-5200 measurement system). Figure 8 and Figure 9 show that the material reactions of the specimens Flat-20-1, Flat-15-1, and Flat-10-1 were almost identical, which was expected due to the identical test conditions. This observation is further confirmed by the mass loss and recession values of the specimens, which are relatively close to each other, as shown in Table 4. The specimens Flat-20-1, Flat-15-1, and Flat-10-1 exhibited an average recession rate of 0.05 mm/s.

### 3.2. Flat-Faced Specimen Transient Tests

After flat-faced specimen stationary tests, three flat-faced specimens, namely, Flat-20-2, Flat-15-2, and Flat-10-2, were subjected to transient tests (6.25↔9.4 MW/m^2^, ~108 s). As mentioned earlier, since pyrometer stagnation point temperature measurement was not possible for the transient tests; only the internal temperatures at TC1, TC2, and TC3 locations were measured for each specimen during the transient tests.

**Figure 10:**
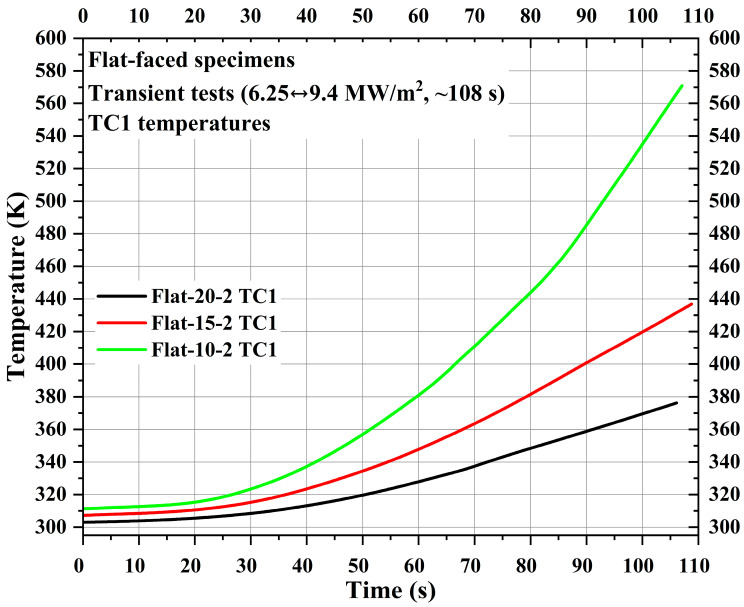
Flat-faced specimen transient test TC1 temperatures.

Figure 10 shows TC1 measurements for the specimens Flat-20-2, Flat-15-2, and Flat-10-2. The locations of TC1, TC2, and TC3 were identical to the corresponding flat-faced specimen used for stationary tests. Figure 10 shows that in flat-faced specimen transient tests, similar to flat-faced specimen stationary tests, the TC1 temperature increased more exponentially as the measuring location moved closer to the specimen stagnation point. Comparing TC1 temperatures at the 100 s mark, the difference between the specimens Flat-10-2 and Flat-15-2 is approximately 2-fold greater than the difference between the specimens Flat-15-2 and Flat-20-2. This confirms the steeper rise of the TC1 temperature in specimen Flat-10-2. Also, as the TC1 measuring location moved 5 mm closer to the stagnation point, the difference between the initial and final TC1 temperatures increased almost 2-fold, meaning that the initial and final TC1 temperature difference value for the specimen Flat-20-2 is ~74 K, whereas the difference value for the specimen Flat-15-2 is ~130 K and difference value for the specimen Flat-10-2 is ~260 K.

**Figure 11:**
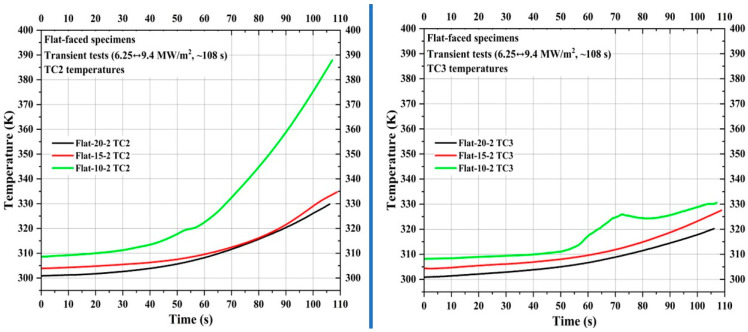
Flat-faced specimen transient test TC2 and TC3 temperatures.

Figure 11 shows TC2 and TC3 measurements of the specimens Flat-20-2, Flat-15-2, and Flat-10-2. Similar to the TC1 temperature of Specimen Flat-10-2, its TC2 temperature also exhibited a steep exponential increase compared to the TC2 temperatures of the other two flat-faced transient specimens. For specimen Flat-10-2, the difference value between the initial and final TC2 temperatures is approximately 2.6-fold greater than the average difference value between the initial and final TC2 temperatures of specimens Flat-20-2 and Flat-15-2. For reasons unknown, the TC3 temperature of specimen Flat-10-2 showed a steep rise around the 55 s mark, followed by a sudden decrease around the 72 s mark, and later followed a trend similar to the other two specimens. This kind of sudden rise and decrease was not observed in the TC3 temperatures of specimens Flat-20-2 and Flat-15-2. The average initial and final TC3 temperature difference value for three flat-faced transient specimens is ~21 K.

**Figure 12:**
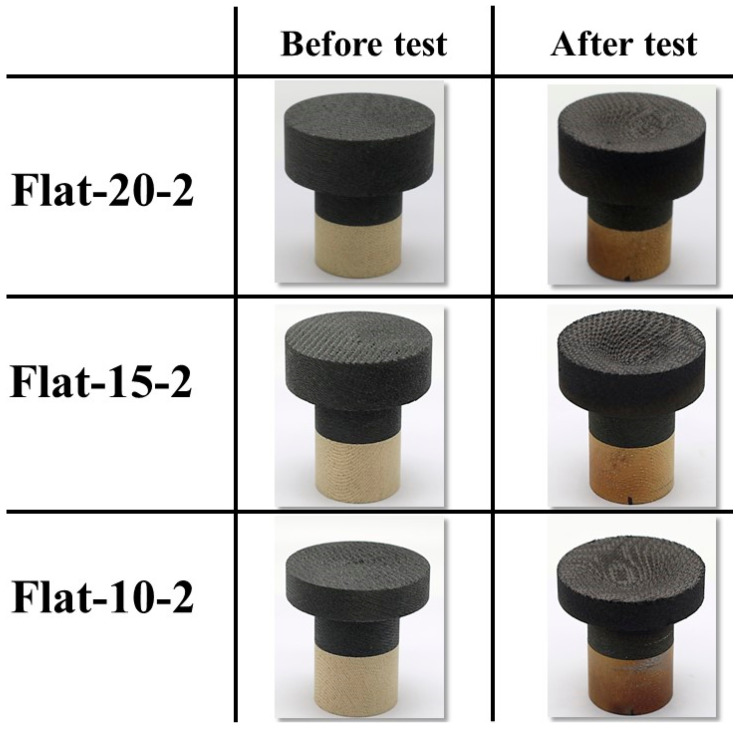
Flat-faced specimen transient tests (6.25↔9.4 MW/m^2^, ~108 s) before- and after-test images.

**Figure 13:**
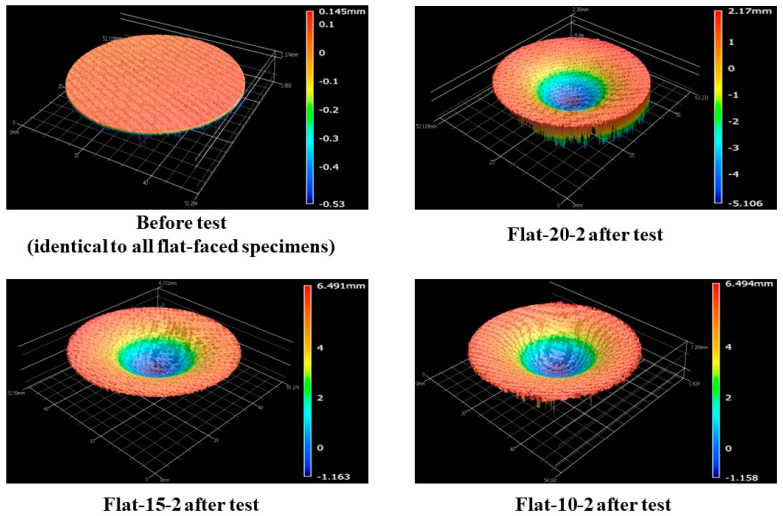
Flat-faced specimen transient test (6.25↔9.4 MW/m^2^, ~108 s)-exposed surface morphology changes.

Figure 12 shows the before- and after-test images of the specimens Flat-20-2, Flat-15-2, and Flat-10-2. Figure 13 shows the after-test-exposed surface 3-D morphologies of the specimens Flat-20-2, Flat-15-2, and Flat-10-2, compared with a before-test flat-faced specimen surface 3-D morphology. Figure 12 and Figure 13 show identical levels of ablation on all three specimen surfaces. Table 5 shows the mass loss and recession values for the flat-faced specimen transient tests, where the values are relatively close to each other. Comparing Table 5 with Table 4 (flat-faced stationary specimens) reveals that the average mass loss value of flat-faced transient specimens is approximately 2.3-fold greater than that of the flat-faced stationary specimens. It also shows that the average recession value of the flat-faced transient specimens is approximately 3.5-fold greater than that of the flat-faced stationary specimens. In the case of the specimens Flat-20-2, Flat-15-2, and Flat-10-2, the average recession rate was 0.07 mm/s.

### 3.3. Hemispherical-Faced Specimen Transient Tests

After flat-faced specimen transient tests, transient tests (6.25↔9.4 MW/m^2^, ~108 s) of three hemispherical-faced specimens, namely, Hemi-20-2, Hemi-15-2, and Hemi-10-2 were carried out.

First, the transient test of specimen Hemi-20-2 was conducted using the type-1 specimen holder. Upon a close visual inspection of the video files recorded during the test, it was observed that the curved shape of specimen Hemi-20-2’s exposed surface directed the test flow onto the sides of the specimen holder, and the resulting mechanical stress led to the breakage of the holder and the specimen. To avoid such issues and also to reduce the unwanted heat load in the lateral directions of the specimens, a modified specimen holder (type-4) was fabricated and used for all subsequent tests of hemispherical-faced specimens.

**Figure 14:**
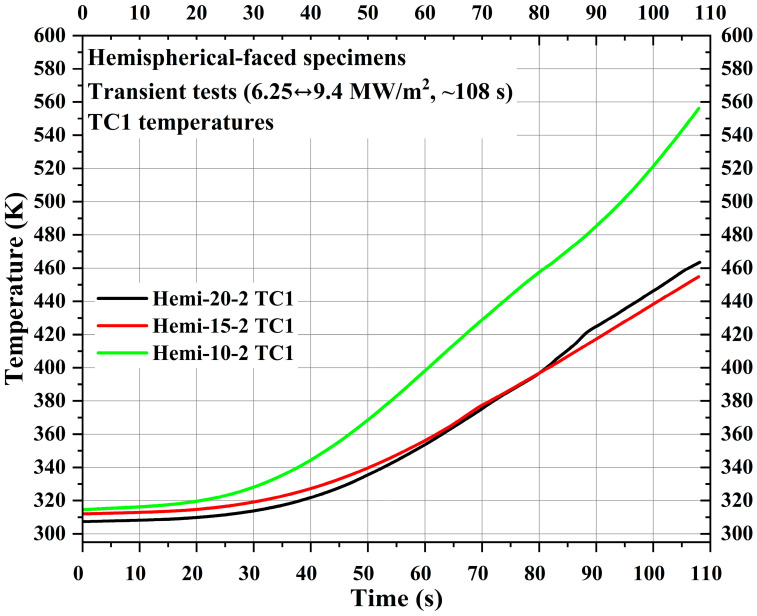
Hemispherical-faced specimen transient test TC1 temperatures.

Figure 14 shows TC1 measurements for the specimens Hemi-20-2, Hemi-15-2, and Hemi-10-2. The locations of TC1 from the stagnation point in the specimens Hemi-20-2, Hemi-15-2, and Hemi-10-2 were 36.88 mm, 31.88 mm, and 26.88 mm, respectively. In Figure 14, the TC1 temperature values of the specimens Hemi-20-2 and Hemi-15-2 are very close to each other, with the TC1 temperature values of the specimen Hemi-20-2 even increasing above the TC1 temperature values of the specimen Hemi-15-2 near the end of the test duration. This should not be the case, as seen in Figure 10 (flat-faced specimen transient tests TC1 temperatures). The discrepancy was due to the design flaw in the type-1 specimen holder used for the specimen Hemi-20-2, as mentioned earlier, hence the need for modification of the specimen holders. Further comparison between Figure 10 and Figure 14 reveals that the TC1 temperature values of specimen Hemi-10-2 are almost similar to those of specimen Flat-10-2, despite the TC1 location of specimen Hemi-10-2 being 11.88 mm further away from the specimen stagnation point compared to Flat-10-2. This is also true for the specimens Hemi-15-2 and Flat-15-2. The reason for this was observed in the stationary tests of the hemispherical-faced specimens and discussed in Section 3.4.

**Figure 15:**
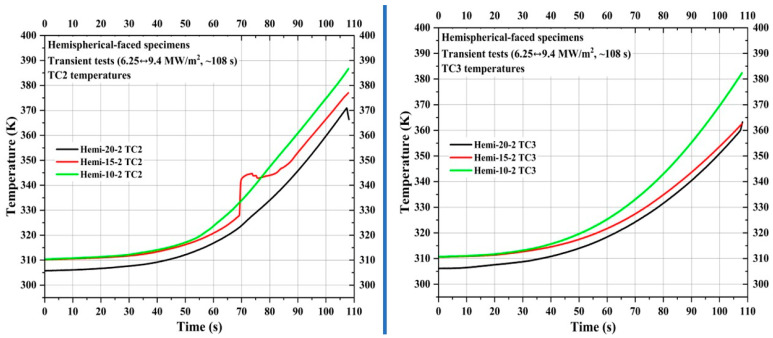
Hemispherical-faced specimen transient test TC2 and TC3 temperatures.

Figure 15 shows TC2 and TC3 measurements of the specimens Hemi-20-2, Hemi-15-2, and Hemi-10-2. TC2 locations of the specimens Hemi-20-2, Hemi-15-2, and Hemi-10-2 were 46.88 mm, 41.88 mm, and 36.88 mm, respectively, from the specimen stagnation point, while TC3 locations were 56.88 mm, 51.88 mm, and 46.88 mm, respectively, from the specimen stagnation point. Figure 15 shows that the TC2 temperature values of specimen Hemi-10-2 are closer to those of specimen Flat-10-2 (seen in Figure 11), despite being 11.88 mm further away from the specimen stagnation point than the TC2 of specimen Flat-10-2. Even though, in Figure 11, the rises seen in the TC2 temperature values of specimens Flat-20-2 and Flat-15-2 are not as steep as the rise in the TC2 temperature for specimen Flat-10-2, in Figure 15, all three hemispherical-faced transient specimens show similar trends in TC2 temperature values. For reasons unknown, the TC2 temperature of specimen Hemi-15-2 briefly increased above the TC2 temperature of specimen Hemi-10-2 and then fell below it. Figure 15 also shows that the TC3 temperature values of the hemispherical-faced transient specimens increased higher than those of the flat-faced transient specimens, with trends similar to TC2 temperature values.

**Figure 16:**
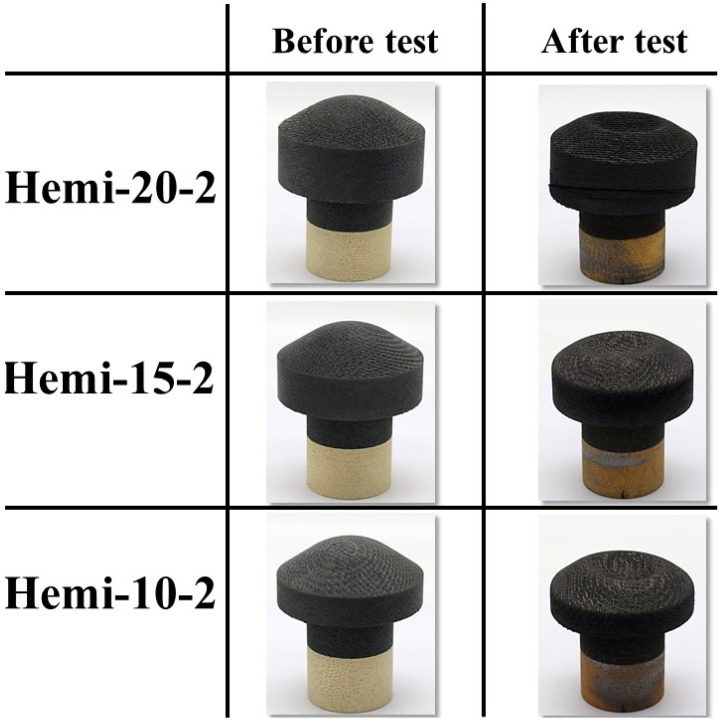
Hemispherical-faced specimen transient tests (6.25↔9.4 MW/m^2^, ~108 s) before- and after-test images.

**Figure 17:**
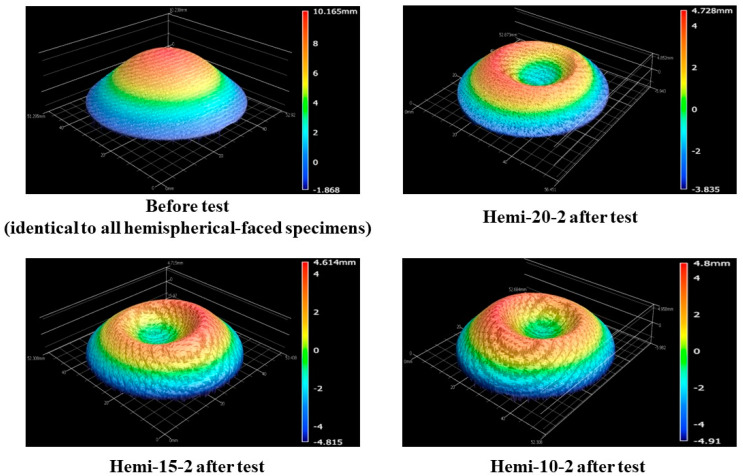
Hemispherical-faced specimen transient test (6.25↔9.4 MW/m^2^, ~108 s)-exposed surface morphology changes.

Figure 16 shows the before- and after-test images of the specimens Hemi-20-2, Hemi-15-2, and Hemi-10-2. Figure 17 shows the after-test-exposed surface 3-D morphologies of the specimens Hemi-20-2, Hemi-15-2, and Hemi-10-2, compared with a before-test hemispherical-faced specimen surface 3-D morphology. Table 6 shows the mass loss and recession values of the specimens Hemi-20-2, Hemi-15-2, and Hemi-10-2. Figure 16 and Figure 17 and Table 6 indicate that the surface reactions of the hemispherical-faced transient test specimens to the plasma test flow were similar. Figure 16 also shows the damage that occurred (broken due to a combination of mechanical stress and relatively high lateral thermal load) in the specimen Hemi-20-2 due to the design flaw in the specimen holder. It is interesting to note that the angle of the crack developed in the specimen Hemi-20-2 was almost the same as the lamination angle of the carbon-phenolic material, i.e., 30°. This suggests that the specimen was broken along the interface between two laminates. Also, since only the TC1 temperature values of specimen Hemi-20-2 (measured inside the specimen’s carbon-phenolic recession layer) were higher than the TC1 temperature values of specimen Hemi-15-1 near the end of the test duration, while the TC2 and TC3 temperatures of specimen Hemi-20-2 were lower than those of the specimen Hemi-15-1 throughout the test duration, this indicates that increased lateral thermal loads due to the specimen holder design flaw and its eventual damage were contained mostly within the carbon-phenolic recession layer of the specimen Hemi-20-2.

Table 6 shows that both the mass loss and recession values of the hemispherical-faced transient specimens are approximately 1.3-fold greater than those of the flat-faced transient specimens, and the average recession rate of the hemispherical-faced transient specimens was 0.09 mm/s.

### 3.4. Hemispherical-Faced Specimen Stationary Tests

Finally, three hemispherical-faced specimens, namely, Hemi-20-1, Hemi-15-1, and Hemi-10-1, were subjected to stationary tests (7.5 Mw/m^2^, ~40 s). As explained earlier, the modified type-4 specimen holder was used for these tests.

**Figure 18:**
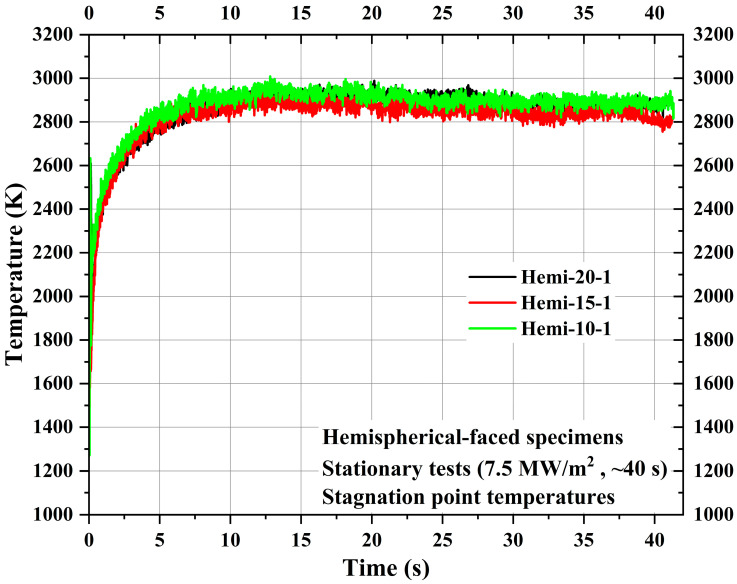
Hemispherical-faced specimen stationary test stagnation point temperatures.

Figure 18 shows the comparison of the stagnation point temperatures of the specimens Hemi-20-1, Hemi-15-1, and Hemi-10-1. Figure 18 shows that all three hemispherical-faced stationary specimens exhibited similar stagnation point temperatures during the tests. The maximum stagnation point temperature values measured for the specimens Hemi-20-1, Hemi-15-1, and Hemi-10-1 are 2989.05 K, 2933.25 K, and 3009.15 K, respectively. On comparing with the average maximum stagnation point temperature of all three flat-faced stationary specimens, the average value of all three hemispherical-faced specimens is ~350 K higher. The only reason for this higher stagnation point temperature is the shape of the exposed surface, as all other parameters, such as test conditions and materials, were identical.

**Figure 19:**
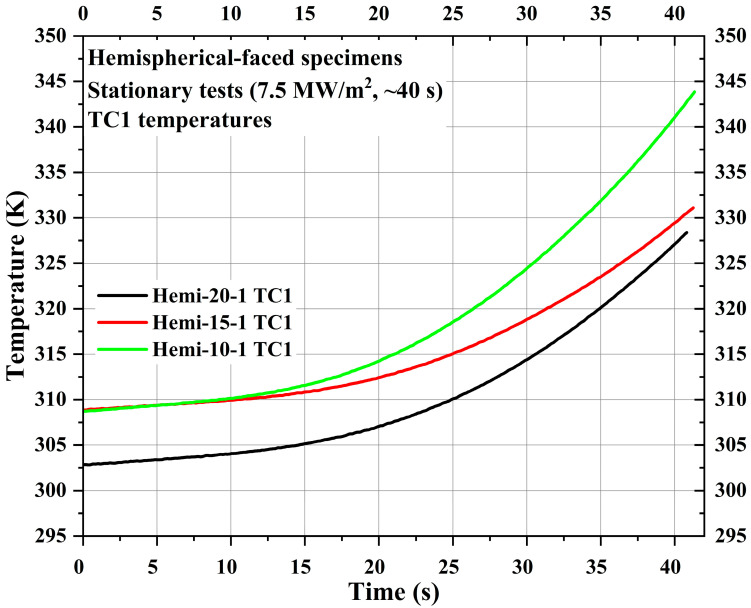
Hemispherical-faced specimen stationary test TC1 temperatures.

**Figure 20:**
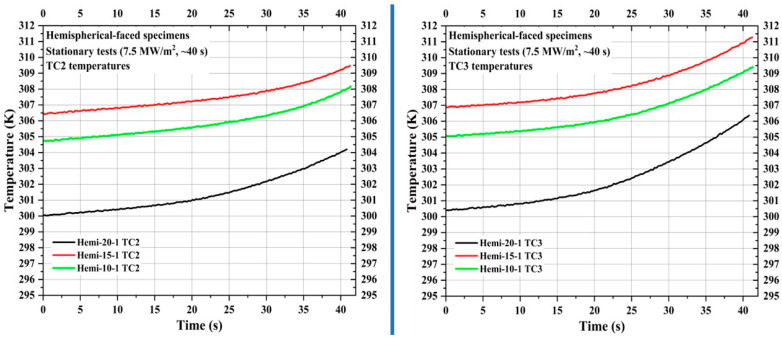
Hemispherical-faced specimen stationary test TC2 and TC3 temperatures.

Figure 19 shows TC1 temperatures of the specimens Hemi-20-1, Hemi-15-1, and Hemi-10-1, whereas Figure 20 shows the TC2 and TC3 temperatures. The locations of TC1, TC2, and TC3 in hemispherical-faced stationary specimens were identical to the corresponding hemispherical-faced specimens used for transient tests. Though the locations of TC1 in the specimens Hemi-20-1, Hemi-15-1, and Hemi-10-1 were 11.88 mm further away from the stagnation point when, respectively, compared with the specimens Flat-20-1, Flat-15-1, and Flat-10-1 (flat-faced stationary specimens), the TC1 temperature values of the hemispherical-faced stationary specimens are seen elevated. For a similar comparison, the TC2 and TC3 temperature values of the hemispherical-faced specimens are also seen elevated.

In Figure 20, it can be seen that the TC2 and TC3 temperature values of specimen Hemi-15-1 are higher than the corresponding temperature values of specimen Hemi-10-1, even though the specimen Hemi-15-1 TC1 temperatures were lower than the specimen Hemi-10-1 TC1 temperatures.

In all conducted tests, the internal temperature data showed that each specimen’s internal temperatures were higher than the room temperature of 293.15 K (20 °C) moments before their exposure to the plasma test flow. The reason for this is an increase in ambient temperature inside the PWT test chamber caused by the generation of the plasma test flow, as the plasma test flow temperature was measured previously as 4350 K [35]. In the case of specimen Hemi-15-1, there was a considerable delay in its exposure to the plasma test flow after the start of plasma generation. This delay led to a significant increase in its internal temperatures prior to the test due to ambient temperature rise, causing the TC2 and TC3 temperatures of specimen Hemi-15-1 to be higher than those of specimen Hemi-10-1 at the start of the test, which continued throughout the test, which was not seen in other results. When compared, it can be seen in most tests that the initial temperatures at TC1, TC2, and TC3 (measured at the start of the tests, i.e., at 0 s) were inversely proportional to the recession layer thickness and their distance from the specimen’s stagnation point. This indicates that the recession layer thickness and measurement location also influence the specimen’s internal temperatures before its exposure to the plasma test flow.

**Figure 21:**
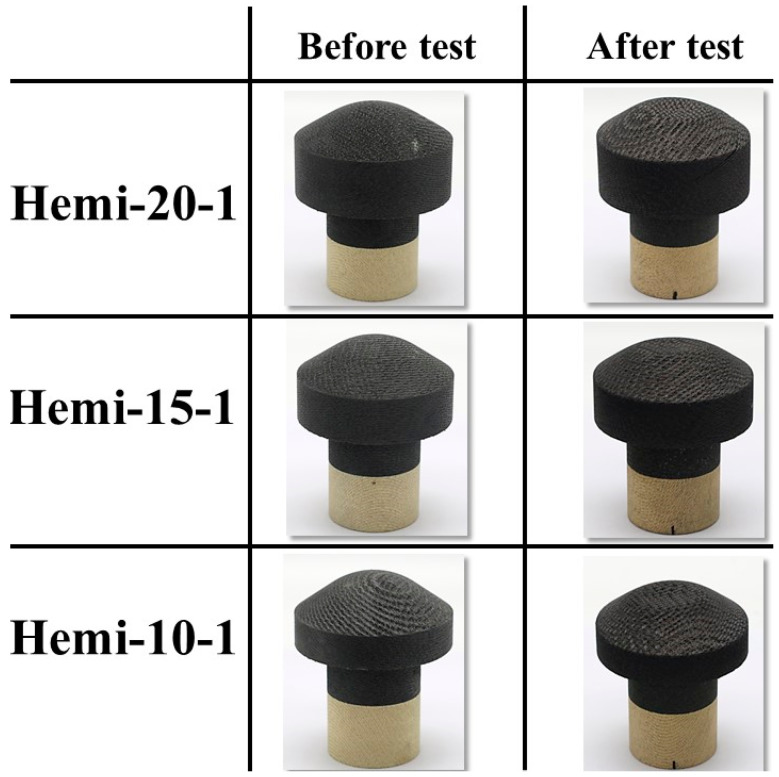
Hemispherical-faced specimen stationary tests (7.5 MW/m^2^, ~40 s), before- and after-test images.

**Figure 22:**
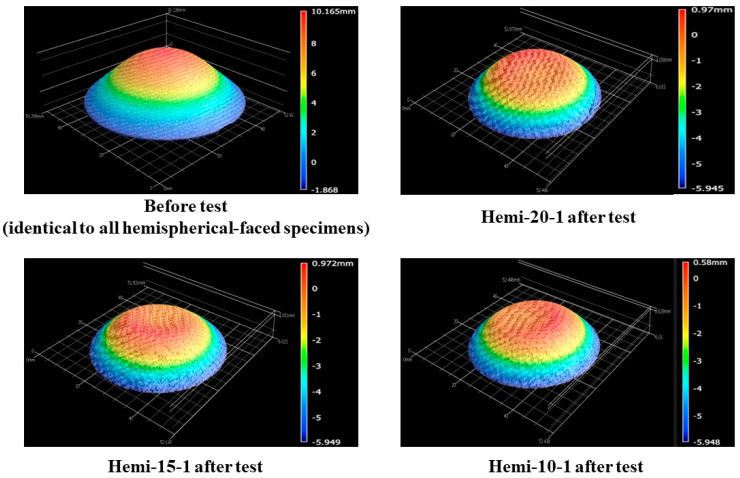
Hemispherical-faced specimen stationary test (7.5 MW/m^2^, ~40 s)-exposed surface morphology changes.

Figure 21 shows the before- and after-test images of the hemispherical-faced stationary specimens. Figure 22 shows the after-test-exposed surface 3-D morphologies of the hemispherical-faced stationary specimens, compared with a before-test hemispherical-faced specimen surface 3-D morphology. Table 7 shows the mass loss and recession values of the hemispherical-faced stationary specimens. As observed in all other tests, changes occurred on the exposed surfaces of all three hemispherical-faced stationary specimens due to the tests being similar, as indicated by Figure 21 and Figure 22. This is also confirmed by closer values of mass loss and recession seen in Table 7. When compared with flat-faced stationary specimens, the average values of mass loss and recession of the hemispherical-faced stationary specimens are approximately 1.4- and 1.5-fold higher, respectively. This is consistent with the comparison results of transient specimens. The average recession rate of the hemispherical-faced stationary specimens was 0.07 mm/s, the same as the flat-faced transient specimens.

The surface temperature increase caused by the hemispherical exposed surface shape is considered responsible for the elevated internal temperatures observed in the hemispherical-faced specimens. This is one of the crucial parameters that need to be considered in the actual design of a spacecraft heat shield, as the shape of the heat shield is more curved near its front and becomes flatter as the radial distance increases. Also, based on the comparison of mass loss and recession values from all tests, it can be concluded that under identical test conditions, hemispherical-faced specimens, solely due to their exposed surface shape, experience approximately 1.4-fold more mass loss and recede approximately 1.4-fold more when compared to flat-faced specimens. This means that the front part (nose-cone) of the spacecraft heat shield will ablate more than the surface locations further away on the heat shield, even when subjected to identical heat flux. These two aspects of how, both the surface temperature (and its influence on internal temperatures) and the material loss change according to the ablator shape, will be helpful in the effective design of the spacecraft heat shield and the internal metallic structure.

It should also be noted that for all tests and specimens, TC2 and TC3 temperatures were well below the design limit of 453.15 K (i.e., 180 °C).

## 4. Conclusions

The CP/SP dual-layer ablator was tested under various structural configurations to gain a better understanding of its thermal behavior. The ablator specimens were tested using a 0.4 MW supersonic arc-jet PWT under two different test flux conditions: (1) stationary test condition (7.5 MW/m^2^, ~40 s) and (2) interplanetary re-entry heat flux trajectory simulating transient test condition (6.25↔9.4 MW/m^2^, ~108 s). The tested specimens were fabricated with two different surface shapes, which were exposed to the plasma test flow: flat-faced and hemispherical-faced. The hemispherical-faced specimens were specifically designed to replicate the nose-cone or front-section of a re-entry spacecraft. The test specimens had varying carbon-phenolic recession layer thicknesses and a constant silica-phenolic insulating layer thickness, and in total, 12 specimens were tested.

The stagnation point temperatures measured during the stationary tests indicated that the hemispherical-faced specimens had higher stagnation point temperatures, approximately 350 K higher than the flat-faced specimens, solely due to their exposed surface shape. The average maximum stagnation point temperatures of the hemispherical-faced and flat-faced specimens during stationary tests were around 2977 K and 2628 K, respectively.

The internal temperatures measured within the carbon-phenolic recession layer of the specimens indicated that as the measuring location moves closer to the stagnation point, the internal temperatures inside the recession layer rise more exponentially. The recession-layer internal temperatures of hemispherical-faced specimens were higher than those of flat-faced specimens because of the higher stagnation-point temperatures. In all but one case, the internal temperatures measured at the recession layer and insulating layer interaction, as well as inside the insulating layer of the specimens, were directly proportional to the recession layer’s internal temperature, which, in turn, depends on the recession layer thickness and exposed surface shape. The behavioral characterization of the internal temperature will be useful in determining the ratio between the recession layer and insulating layer, thereby sizing the CP/SP ablator for the actual spacecraft heat shield design. For all specimen tests, irrespective of carbon-phenolic recession layer thickness and shape, the internal temperatures measured inside the silica-phenolic insulating layers were below 453.15 K (i.e., 180 °C) during the test durations, thus meeting the design criteria set for the CP/SP ablator. At the end of the stationary tests, the internal temperatures of the insulating layers for both flat-faced and hemispherical-faced specimens were around 300~311 K. At the end of the transient tests, hemispherical-faced specimens exhibited an average internal insulating layer temperature of approximately 369 K, while flat-faced specimens reached an average internal insulating layer temperature of about 326 K.

The hemispherical-faced specimens exhibited approximately 1.4-fold times more mass loss and recession than the flat-faced specimens. The average recession rate of stationary flat-faced specimens was 0.05 mm/s; for both transient flat-faced specimens and stationary hemispherical-faced specimens, it was 0.07 mm/s; and for transient hemispherical-faced specimens, it was 0.09 mm/s. This information will be useful to determine the overall design of the spacecraft heat shield. Thermal structure analysis that can analyze and predict the temperature of the carbon-phenolic/silica phenolic dual-layer ablator specimen using the temperature data inside the specimen at various heat fluxes derived from this study will be conducted in the near future.

In this study, an experimental study was conducted on a scaled specimen. There was also a limit to not being able to test the entire profile of the Earth’s re-entry heat flux. In the future, we hope that a test evaluation of the real-size model will be conducted under the conditions of the entire profile of the re-entry heat flux.

## Figures and Tables

**Table 1:** PWT operating conditions.

Operating Condition	Value
Working gas flow rate	4.14 g/s
Air percentage in working gas	95.05%
Argon percentage in working gas	4.95%
Applied total current	140 A
Applied total voltage	578.25 V
Operated total torch power	80.96 kW

**Table 2:** Specimen test conditions.

Test Condition	Heat Flux (MW/m^2^)	Distance from the Torch Nozzle Exit (mm)	Duration (s)
Stationary	7.5	170	~40
Transient *	from 6.25 ^±^ to 9.4	180 to 120	50
	9.4	120	~8 ^€^
	from 9.4 to 6.25 ^±^	120 to 180	50

* Continuous exposure to the test flow, a total of ~108 s. ^±^ Empirically estimated from experimental values measured using the Gardon gauge. ^€^ Average displacement mechanism direction reset time.

**Table 3:** Specimen specifications.

No.	Specimen	Exposed Surface Shape and Test Condition	Specimen Length or Thickness (mm)	Carbon-Phenolic Recession Layer Thickness (mm)	Thermocouple Measurement Location from Specimen Stagnation Point (mm)		
					TC1	TC2	TC3
1	Flat-20-1	Flat and stationary	55	35	25	35	45
2	Flat-20-2	Flat and transient					
3	Flat-15-1	Flat and stationary	50	30	20	30	40
4	Flat-15-2	Flat and transient					
5	Flat-10-1	Flat and stationary	45	25	15	25	35
6	Flat-10-2	Flat and transient					
7	Hemi-20-1	Hemispherical and stationary	66.88	46.88	36.88	46.88	56.88
8	Hemi-20-2	Hemispherical and transient					
9	Hemi-15-1	Hemispherical and stationary	61.88	41.88	31.88	41.88	51.88
10	Hemi-15-2	Hemispherical and transient					
11	Hemi-10-1	Hemispherical and stationary	56.88	36.88	26.88	36.88	46.88
12	Hemi-10-2	Hemispherical and transient					

For all specimens, silica-phenolic insulating layer thickness = 20 mm. For all specimens, TC1, TC2, and TC3 locations from the bottom surface were 30 mm, 20 mm, and 10 mm, respectively.

**Table 4:** Flat-faced specimen stationary tests mass loss and recession.

Specimen	Test Condition *	Mass Loss (g)	Recession (mm)
Flat-20-1	7.5 MW/m^2^, 41.39 s	5.55	2.24
Flat-15-1	7.5 MW/m^2^, 41.24 s	5.26	1.99
Flat-10-1	7.5 MW/m^2^, 41.29 s	4.65	1.96

Mass loss = specimen before test mass − specimen after test mass. Recession = specimen before test length − specimen after test length. * Actual test duration was confirmed using pyrometer data and test video files.

**Table 5:** Flat-faced specimen transient tests mass loss and recession.

Specimen	Test Condition *	Mass Loss (g)	Recession (mm)
Flat-20-2	6.25↔9.4 MW/m^2^, 106.12 s	12.35	6.73
Flat-15-2	6.25↔9.4 MW/m^2^, 108.8 s	12.32	7.66
Flat-10-2	6.25↔9.4 MW/m^2^, 107.8 s	11.57	7.55

Mass loss = specimen before test mass − specimen after test mass. Recession = specimen before test length − specimen after test length. * Actual test duration was confirmed using test video files.

**Table 6:** Hemispherical-faced specimen transient tests mass loss and recession.

Specimen	Test Condition *	Mass Loss (g)	Recession (mm)
Hemi-20-2	6.25↔9.4 MW/m^2^, 108.17 s	17.18	9.06
Hemi-15-2	6.25↔9.4 MW/m^2^, 107.96 s	15.55	10.01
Hemi-10-2	6.25↔9.4 MW/m^2^, 107.99 s	15.09	9.56

Mass loss = specimen before test mass − specimen after test mass. Recession = specimen before test length − specimen after test length. * Actual test duration was confirmed using test video files.

**Table 7:** Hemispherical-faced specimen stationary tests mass loss and recession.

Specimen	Test Condition *	Mass Loss (g)	Recession (mm)
Hemi-20-1	7.5 MW/m^2^, 40.81 s	7.47	2.58
Hemi-15-1	7.5 MW/m^2^, 41.25 s	7.38	3.35
Hemi-10-1	7.5 MW/m^2^, 41.34 s	6.92	3.16

Mass loss = specimen before test mass − specimen after test mass. Recession = specimen before test length − specimen after test length. * Actual test duration was confirmed using pyrometer data and test video files.

## Data Availability

The data will be made available on request from the corresponding author.

## Nomenclature

CP	carbon–phenolic
CPBAM	carbon–phenolic-based ablative material
ESA	European Space Agency
HVOF	high-velocity oxygen fuel
JBNU	Jeonbuk National University
MC-CFRP	MUSES-C carbon fiber-reinforced polymer
NASA	National Aeronautics and Space Administration
NI	National Instruments
PICA	phenolic impregnated carbon ablator
PWT	plasma wind tunnel
RCC	reinforced carbon-carbon
SP	silica–phenolic
SPOF	single point of failure
TC	thermocouple
TPS	thermal protection system
USB	universal serial bus

## References

[1] Laub B. Venkatapathy E Thermal protection system technology and facility needs for demanding future planetary missions. Proceedings of the International Workshop Planetary Probe Atmospheric Entry and Descent Trajectory Analysis and Science.

[2] Pulci G., Tirillò J., Marra F., Fossati F., Bartuli C., Valente T. (2010). Carbon–phenolic ablative materials for re-entry space vehicles: Manufacturing and properties. Compos. Part A Appl. Sci. Manuf..

[3] Curry D.M. Space Shutlle Orbiter Thermal Protection System Design and Flight Experience. Proceedings of the First ESA/ESTEC Workshop on Thermal Protection Systems Noordwijk.

[4] Paglia L., Genova V., Tirillò J., Bartuli C., Simone A., Pulci G., Marra F. (2021). Design of New Carbon-Phenolic Ablators: Manufacturing, Plasma Wind Tunnel Tests and Finite Element Model Rebuilding. Appl. Compos. Mater..

[5] Bessire B.K., Lahankar S.A., Minton T.K. (2015). Pyrolysis of Phenolic Impregnated Carbon Ablator (PICA). ACS Appl. Mater. Interfaces.

[6] Pielichowska K., Paprota N., Pielichowski K. (2023). Fire Retardant Phase Change Materials&mdash;Recent Developments and Future Perspectives. Materials.

[7] Natali M., Kenny J.M., Torre L. (2016). Science and technology of polymeric ablative materials for thermal protection systems and propulsion devices: A review. Prog. Mater. Sci..

[8] Pirrone S.R.M., Agabiti C., Pagan A.S., Herdrich G. (2022). A Fast Thermal 1D Model to Study Aerospace Material Response Behaviors in Uncontrolled Atmospheric Entries. Materials.

[9] Pavlosky J.E., Leger L.G.S. (1974). Apollo Experience Report—Thermal Protection Subsystem.

[10] Davies C., Arcadi M. (2006). Planetary Mission Entry Vehicles Quick Reference Guide.

[11] Yamada T., Inatani Y., Hirai K.C. (2003). Thermal Responses of Ablator for Reentry Capsules with Superorbital Velocity.

[12] Harris R., Stewart M., Koenig W. Thermal Protection Systems Technology Transfer from Apollo and Space Shuttle to the Orion Program. Proceedings of the 2018 AIAA SPACE and Astronautics Forum and Exposition, AIAA SPACE Forum, American Institute of Aeronautics and Astronautics.

[13] Milos F.S., Chen Y.K. (2010). Ablation and Thermal Response Property Model Validation for Phenolic Impregnated Carbon Ablator. J. Spacecr. Rocket..

[14] Canfield A., Koenig J. Development of PAN precursor materials for solid propellant rocket motor nozzles. Proceedings of the 25th Joint Propulsion Conference.

[15] Williams G., Murray J. Status on Replacing Rayon Based Carbon Phenolic Ablatives in the MK-104 Motor. Proceedings of the 44th AIAA/ASME/SAE/ASEE Joint Propulsion Conference & Exhibit, Joint Propulsion Conferences. American Institute of Aeronautics and Astronautics.

[16] Shi S., Wang Y., Jiang T., Wu X., Tang B., Gao Y., Zhong N., Sun K., Zhao Y., Li W. (2022). Carbon Fiber/Phenolic Composites with High Thermal Conductivity Reinforced by a Three-Dimensional Carbon Fiber Felt Network Structure. ACS Omega.

[17] Kaufman J.G. (2016). Fire Resistance of Aluminum and Aluminum Alloys and Measuring the Effects of Fire Exposure on the Properties of Aluminum Alloys.

[18] Paglia L., Tirillò J., Marra F., Bartuli C., Simone A., Valente T., Pulci G. (2016). Carbon-phenolic ablative materials for re-entry space vehicles: Plasma wind tunnel test and finite element modeling. Mater. Des..

[19] Natali M., Torre L., Puri I., Rallini M. (2022). Thermal degradation of phenolics and their carbon fiber derived composites: A feasible protocol to assess the heat capacity as a function of temperature through the use of common DSC and TGA analysis. Polym. Degrad. Stab..

[20] Milos F.S., Chen Y.-K., Mahzari M. (2018). Arcjet Tests and Thermal Response Analysis for Dual-Layer Woven Carbon Phenolic. J. Spacecr. Rocket..

[21] Chinnaraj R.K., Kim Y.C., Choi S.M. (2023). Thermal Ablation Experiments of Carbon Phenolic and SiC-Coated Carbon Composite Materials Using a High-Velocity Oxygen-Fuel Torch. Materials.

[22] Chinnaraj R.K., Kim Y.C., Choi S.M. (2023). Arc-Jet Tests of Carbon-Phenolic-Based Ablative Materials for Spacecraft Heat Shield Applications. Materials.

[23] Chinnaraj R.K. (2022). Supersonic High Temperature Flow Diagnosis and Material Ablation Experiments Using the HVOF System. Ph.D. Dissertation.

[24] Chinnaraj R.K., Hong S.M., Kim H.S., Choi S.M. (2023). Ablation Experiments of High-Temperature Materials (Inconel, C–C and SiC) Using a High-Velocity Oxygen-Fuel Torch. Int. J. Aeronaut. Space Sci..

[25] Drescher O., Hörschgen-Eggers M., Pinaud G., Podeur M. Cork based thermal protection system for sounding rocket applications—Development and fight testing. Proceedings of the 23rd ESA Symposium on European Balloon and Rocket Programmes and related Research.

[26] Paixão S., Peixoto C., Reinas M., Carvalho J. (2022). RETALT_TPS design and manufacturing. CEAS Space J..

[27] Hyman T.S. (1977). Moldable Cork Ablation Material.

[28] Shi S., Liang J., Yi F., Fang G. (2012). Modeling of one-dimensional thermal response of silica-phenolic composites with volume ablation. J. Compos. Mater..

[29] Zhou L., Sun X., Chen M., Zhu Y., Wu H. (2019). Multiscale modeling and theoretical prediction for the thermal conductivity of porous plain-woven carbonized silica/phenolic composites. Compos. Struct..

[30] Zibitsker A., Berreby M., Michaels D., Shilav R., Frisman I. (2021). Ultrasonic Temperature Compensating Method for Tracking Decomposition Front in Silica-Phenolic Thermal Protection Material. J. Thermophys. Heat Transf..

[31] Aravind Jithin A.J., Sushanta KPanigrahi Sasikumar P., Shreedhar KRao Shabeeb Ali T.K., Krishnakumar G. (2022). Thermophysical properties of hybrid silica phenolic ablative composite: Theoretical and experimental analysis. Polym. Compos..

[32] Jithin AJ A., Panigrahi S.K., Sasikumar P., Rao K.S., Krishnakumar G. (2022). Ablative properties, thermal stability, and compressive behaviour of hybrid silica phenolic ablative composites. Polym. Degrad. Stab..

[33] Tran P., Paulat J.C., Boukhobza P. (2007). Re-entry Flight Experiments Lessons Learned—The Atmospheric Reentry Demonstrator.

[34] Bouilly J.-M. (2005). Thermal Protection of the Huygens Probe During Titan Entry: Last Questions. 2nd International Planetary Probe Workshop.

[35] Chinnaraj R.K., Oh P.Y., Shin E.S., Hong B.G., Choi S.M. (2019). Mach Number Determination in a High-Enthalpy Supersonic Arc-Heated Plasma Wind Tunnel. Int. J. Aeronaut. Space Sci..

[36] Tekna Plasma Systems Inc. 0.4 MW Class Enhanced Huels Type Plasma System. Operating Manual—System 93.

[37] Loehle S., Zander F., Eberhart M., Hermann T., Meindl A., Massuti-Ballester B., Leiser D., Hufgard F., Pagan A.S., Herdrich G. (2022). Assessment of high enthalpy flow conditions for re-entry aerothermodynamics in the plasma wind tunnel facilities at IRS. CEAS Space J..

[38] Kuzenov V.V., Ryzhkov S.V., Varaksin A.Y. (2023). Computational and Experimental Modeling in Magnetoplasma Aerodynamics and High-Speed Gas and Plasma Flows (A Review). Aerospace.

[39] Hirai K., Nakazato A., Yano H., Kawazone K., Koyanagi J., Yamada K. (2019). Ablative Performance of High Density Carbon Phenolic after Cold Soak Exposure. Trans. Jpn. Soc. Aeronaut. Space Sci. Aerosp. Technol. Jpn..

[40] Tran H.K., Johnson C.E., Rasky D.J., Hui F.C.L., Hsu M.-T., Chen T., Chen Y.K., Paragas D., Kobayashi L. (1997). Phenolic Impregnated Carbon Ablators (PICA) as Thermal Protection Systems for Discovery Missions.

[41] Löhle S., Hermann T., Zander F. (2018). Experimental assessment of the performance of ablative heat shield materials from plasma wind tunnel testing. CEAS Space J..

[42] Pagan A., Zuber C., Massuti-Ballester B., Herdrich G., Hald H., Fasoulas S. The Ablation Performance and Dynamics of the Heat Shield Material ZURAM^®^. Proceedings of the 31st International Symposium on Space Technology and Science.

[43] Rouméas R., Pichon T., Lacombe A. (1998). High-Performance Heat Shields for Planetary Entry Systems.

[44] Helber B., Turchi A., Scoggins J.B., Hubin A., Magin T.E. (2016). Experimental investigation of ablation and pyrolysis processes of carbon-phenolic ablators in atmospheric entry plasmas. Int. J. Heat Mass Transf..

[45] Chinnaraj R.K., Hong S.M., Kim H.S., Oh P.Y., Choi S.M. (2020). Ablation Experiments of Ultra-High-Temperature Ceramic Coating on Carbon–Carbon Composite Using ICP Plasma Wind Tunnel. Int. J. Aeronaut. Space Sci..

[46] Auweter-Kurtz M., Hald H., Koppenwallner G., Speckmann H.D. (1996). German experiments developed for reentry missions. Acta Astronaut..

[47] Anderson J.D. (2006). Hypersonic and High-Temperature Gas Dynamics.

